# Notch Pathway Modulation on Bone Marrow-Derived Vascular Precursor Cells Regulates Their Angiogenic and Wound Healing Potential

**DOI:** 10.1371/journal.pone.0003752

**Published:** 2008-11-18

**Authors:** Francisco Caiado, Carla Real, Tânia Carvalho, Sérgio Dias

**Affiliations:** 1 Angiogenesis Laboratory, CIPM, Portuguese Institute of Oncology, Lisbon, Portugal; 2 Instituto Gulbenkian Ciencia, Oeiras, Portugal; 3 Instituto de Medicina Molecular, Lisbon, Portugal; Dresden University of Technology, Germany

## Abstract

Bone marrow (BM) derived vascular precursor cells (BM-PC, endothelial progenitors) are involved in normal and malignant angiogenesis, in ischemia and in wound healing. However, the mechanisms by which BM-PC stimulate the pre-existing endothelial cells at sites of vascular remodelling/recovery, and their contribution towards the formation of new blood vessels are still undisclosed. In the present report, we exploited the possibility that members of the Notch signalling pathway, expressed by BM-PC during endothelial differentiation, might regulate their pro-angiogenic or pro-wound healing properties. We demonstrate that Notch pathway modulates the adhesion of BM-PC to extracellular matrix (ECM) *in vitro* via regulation of integrin alpha3beta1; and that Notch pathway inhibition on BM-PC impairs their capacity to stimulate endothelial cell tube formation on matrigel and to promote endothelial monolayer recovery following wounding *in vitro*. Moreover, we show that activation of Notch pathway on BM-PC improved wound healing *in vivo* through angiogenesis induction. Conversely, inoculation of BM-PC pre-treated with a gamma secretase inhibitor (GSI) into wounded mice failed to induce angiogenesis at the wound site and did not promote wound healing, presumably due to a lower frequency of BM-PC at the wound area. Our data suggests that Notch pathway regulates BM-PC adhesion to ECM at sites of vascular repair and that it also regulates the capacity of BM-PC to stimulate angiogenesis and to promote wound healing. Drug targeting of the Notch pathway on BM-PC may thus represent a novel strategy to modulate neo-angiogenesis and vessel repair.

## Introduction

The vertebrate skin represents a major barrier against external damage. Maintenance of a functional/undamaged skin namely through an efficient cutaneous wound healing is essential. Cutaneous wound healing involves an inflammatory response, formation of granulation tissue, angiogenesis and tissue remodelling [1], [2]. During these processes there is interplay between different cell types or between cells and the extracellular matrix (ECM) which are mediated by chemokines/growth factors and integrins, respectively. [3], [4]. Angiogenesis, the process by which new capillaries are formed, is a fundamental step in wound healing. The formation of new vessels at the wound site allows the inflammatory cells to migrate into the wound, but also supply the oxygen and nutrients necessary to sustain the growth of the granulation tissue and epidermis [5].

Bone marrow derived progenitor cells (BM-PC) with vasculogenesis/angiogenesis potential have been proven essential in a variety of models of post-natal angiogenesis [6]. Despite the heterogeneity of BM-PC populations, it is now accepted that bone marrow derived endothelial progenitor cells (EPC) and bone marrow derived mesenchymal stem cells (MSC) can enhance angiogenesis and promote vascular healing in different models, such as in cutaneous wound healing. Accordingly, it has been shown that BM-PC can improve angiogenesis at the wound site by differentiation and incorporation into mature vessels and production of pro-angiogenic factors [7], [8], [9]. In addition, recruited BM-PC may promote endothelial cell migration and proliferation via the production of IL-8, VEGF, angiopoietin-1 or stromal derived factor-1 (SDF-1), among other factors [10], 11, 12.

The Notch signalling pathway involves the activity of Notch transmembrane receptors 1, 3 and 4, which interact with membrane-bound ligands, Delta1, 2 and 4 and Serrate/Jagged 1 and 2. Ligand binding induces proteolytic cleavage of Notch receptor by a gamma-secretase complex causing the subsequent translocation of the Notch intracellular domain (NICD) to the nucleus, where it will activate the transcription of downstream target genes such as Hes1 (hairy enhancer of split homolog-1) and Hey1 and Hey2 (Hes related protein) [13]. Deficient Notch signalling impairs normal vascular development in the embryo [14], [15], [16], [17], [18]. More recently involvement of the Notch pathway in cutaneous wound healing was demonstrated, since Notch antisense transgenic mice and normal mice treated with gamma-secretase inhibitors have impaired healing due to defective endothelial and keratinocyte cell migration [19]. However the contribution of BM-PC in these settings was not addressed. Considering this, besides regulating arterial/venous fate [20], [21], the role of the N/D pathway in regulating BM-PC differentiation and function during angiogenesis is still elusive.

In the present study, we hypothesized that the Notch pathway might be involved in the communication between recruited BM-PC and endothelial cells during wound healing. To test this hypothesis, we employed gamma-secretase inhibitors to block Notch activity and overexpression of the Notch ligand Dll4 to address how Notch signalling contributes to the function of BM-PC in vitro and during the angiogenic response in cutaneous wound healing.

## Results

### BM-derived progenitors express Delta-Notch members and show evidence for Notch pathway activation *in vitro*

**Figure 1:**
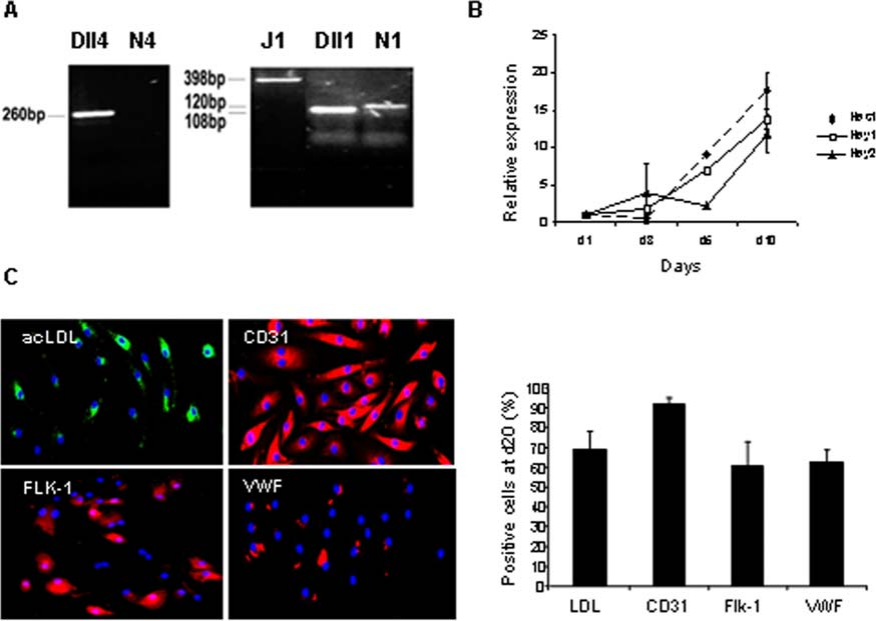
BM-PC express Notch pathway ligands/receptors and show increased expression of notch downstream targets during endothelial differentiation. A. Expression of Notch receptors and ligands in BM-PC was detected by RT-PCR. B. Expression of Notch downstream targets (Hes 1, Hey 1 and 2) was detected at different time points during BM-PC endothelial differentiation by quantitative real-time PCR. C. Representative images (×200) of BM-PC at day 20 of culture showing positivity for endothelial lineage specific markers, acetylated LDL, CD31 , Flk-1 and vWF with DAPI nuclear counterstaining in blue. D. Quantification of BM-PC positive cells for acetylated LDL, CD31 , Flk-1 and VWF after 20 days of culture. Each experiment was performed in triplicate and the mean presented (n = 3).

BM-PC (lin-sca1+) cells were cultured on endothelial-differentiation medium and the expression of Delta-Notch members and their target genes was determined by RT and semi-quantitative (RQ) PCR, throughout endothelial differentiation. As shown in Figure 1A, BM-PC express Delta-like 4, Delta 1 and Notch 1 but do not express Notch 4. The expression of Notch downstream target genes Hes 1, Hey 1 and Hey 2 increases throughout endothelial differentiation (Figure 1B), as the majority of the cultured cells differentiate and acquire endothelial markers and properties (at day 20 of culture, Figure 1C and D and Figure S1). These results suggest that BM-PC give rise to endothelial cells *in vitro* and that this process is accompanied by activation of the Notch signalling pathway and transcription of Notch target genes.

### BM-PC adhesion to ECM *in vitro* is impaired by gamma-secretase inhibition of Notch pathway and integrin alpha3beta1 modulation

**Figure 2:**
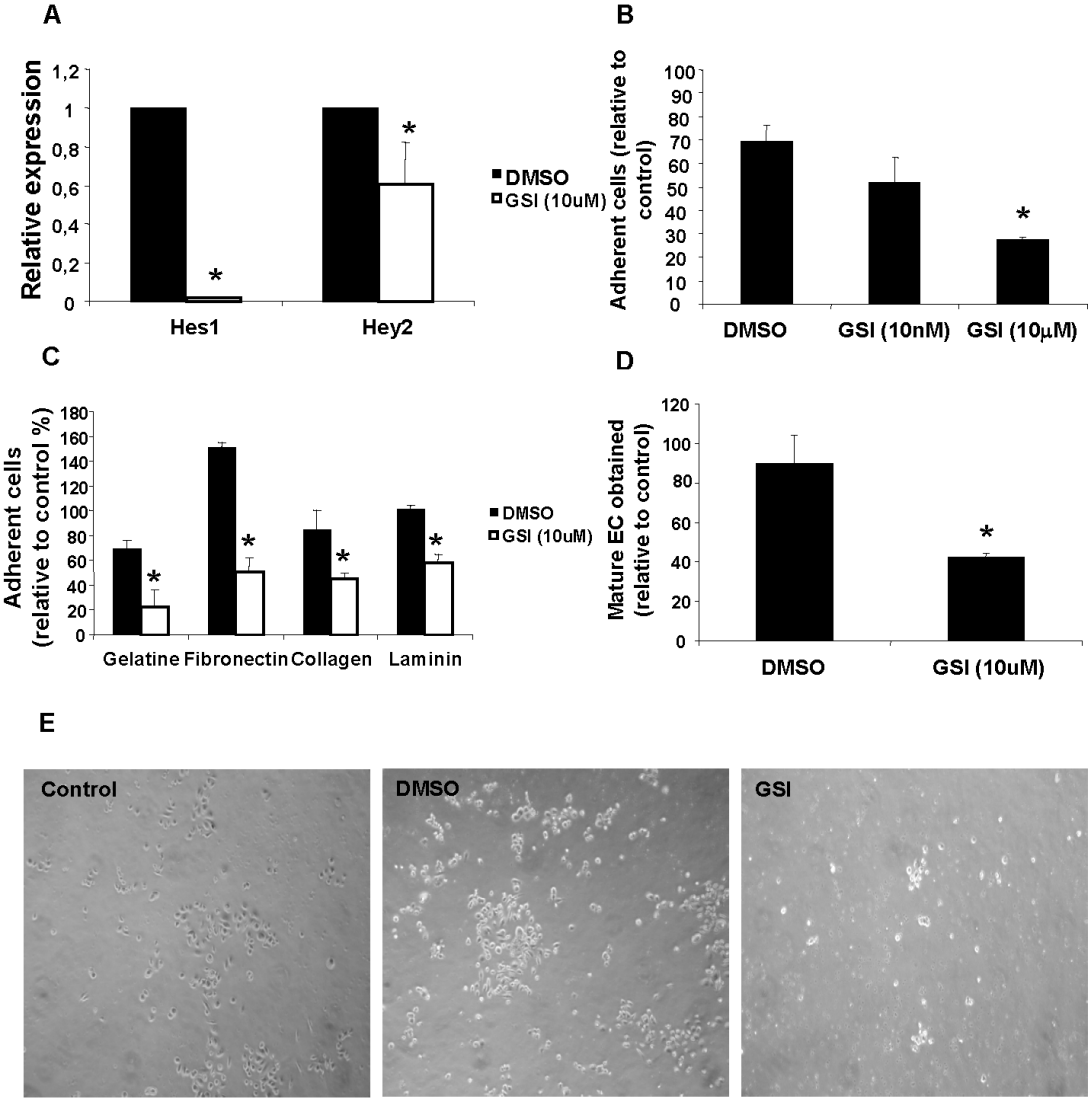
Notch pathway early inhibition impairs BM-PC adhesion and spreading to extracellular matrix, reducing the number of mature cells obtained at the end of the differentiation. A. Expression of Hes 1 and Hey 2 72 h after GSI (10 uM) treatment was detected by RT-PCR. B. Quantification of adherent BM-PC 72 h after treatment with DMSO, GSI at 10 ηM or 10 µM, on 2% gelatin coated wells. C. Quantification of adherent BM-PC 48 h after treatment with DMSO and GSI at 10 µM, on 2% gelatin, fibronectin, collagen or laminin coated wells. D. Quantification of control or GSI BM-PC expressing double EC – lineage specific markers (acLDL/FLK-1 or acLDL/VWF) after 20 days of endothelial differentiation. E. Representative image (100×) of adherent cells under the different conditions. *P<0,05, **P<0,01. Each experiment was performed in triplicate and the mean presented (n = 3).

Having shown activation of Notch signalling on BM-PC under endothelial differentiation conditions *in vitro*, we asked what aspect of the endothelial differentiation process would be affected by inhibiting the Notch pathway. As shown in Figure 2, Notch pathway inhibition by a gamma-secretase-inhibitor (GSI, also known as DAPT) reduced the activation of Notch target genes on BM-PC (Fig 2A), impaired their adhesion to different ECM components (Fig 2B,C,E) and reduced the percentage of endothelial cells obtained under endothelial differentiation conditions *in vitro* (Fig 2D). In contrast, transfection of BM-PC with a constitutively active form of Notch4, to activate the Notch pathway, promoted BM-PC adhesion and endothelial differentiation (Figure S2). Importantly, Notch pathway inhibition with GSI did not affect BM-PC survival or proliferation (data not shown).

**Figure 3:**
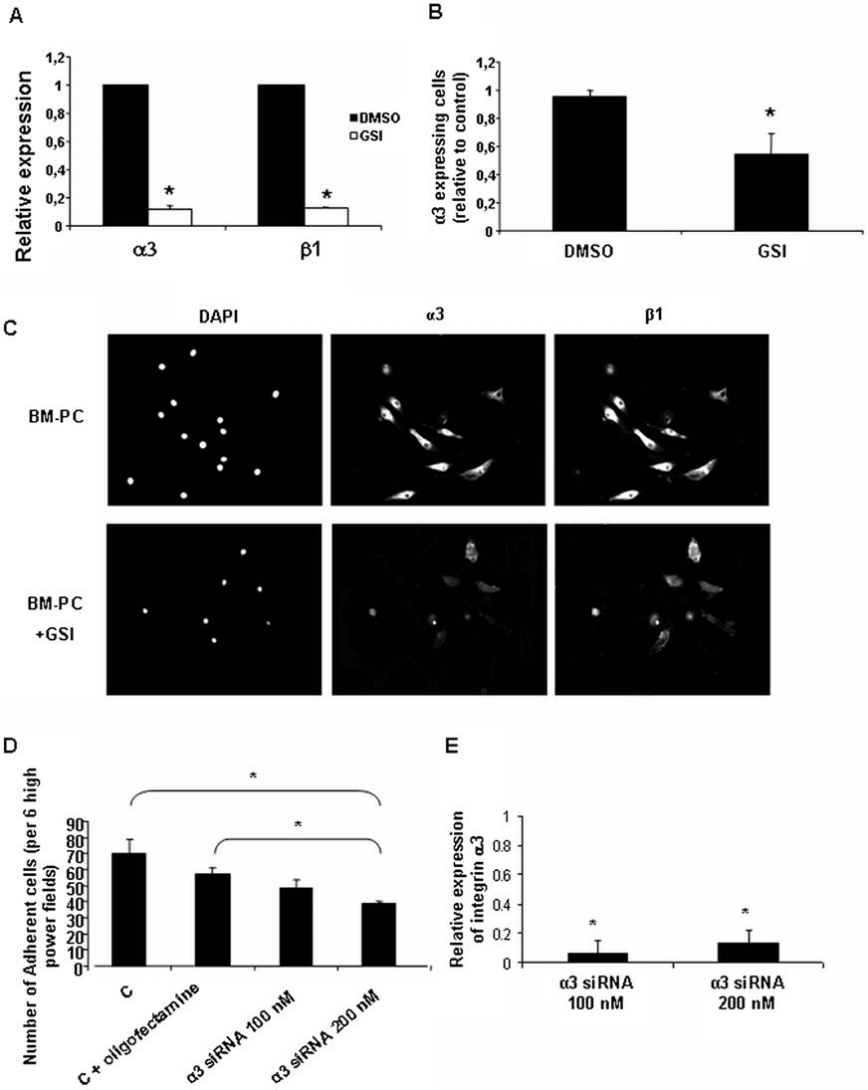
Regulation of the Notch pathway interferes with expression levels of integrin sub-units α3 and β1 in BM-PC. A. Expression of integrin sub-units α3 and β1 determine by real-time PCR on BM-PC after treatment with DMSO or GSI; B. Quantification of α3 expressing BM-PC after treatment with DMSO or GSI. C. Representative images (200×) of adherent BM-PC imunostained for integrin α3 and β1 in control and GSI treated BM-PC. D. Quantification of adherent BM-PC after 48 h of transient transfection with siRNA against integrin sub-unit α3 at concentrations of 100 or 200 ηM. E. Expression of integrin sub-units α3 determine by real-time PCR on BM-PC after transient transfection with siRNA against integrin sub-unit α3 at concentrations of 100 or 200 ηM. *P<0.01. Each experiment was performed in triplicate and the mean presented (n = 3).

Next, we asked if inhibition of the Notch pathway affected BM-PC adhesion to ECM by reducing the expression of specific integrins. As shown in Fig 3 and Table 1, GSI-treated BM-PC showed a significant reduction in the expression of alpha3beta1, while beta 3, alpha5 and alpha v expression levels remained unaffected by GSI treatment. These results demonstrate that the GSI inhibition of BM-PC adhesion to different ECM involves the selective down-regulation of integrins alpha 3 beta 1. Accordingly, siRNA against integrin alpha 3 decreased the number of adherent BM-PC (Fig 3 D,E).

**Table 1:** Integrin sub-units β3, α5 and αv expression on BM-PC after treatment with DMSO or GSI, as determined by FACS analysis.

Integrin	Integrin expressing cells (%)	
	DMSO	GSI
β3	16%	15%
α5	78%	86%
αV	5,71%	7,2%

### Notch pathway inhibition on BM-PC reduces their pro-angiogenic properties *in vitro*

**Figure 4:**
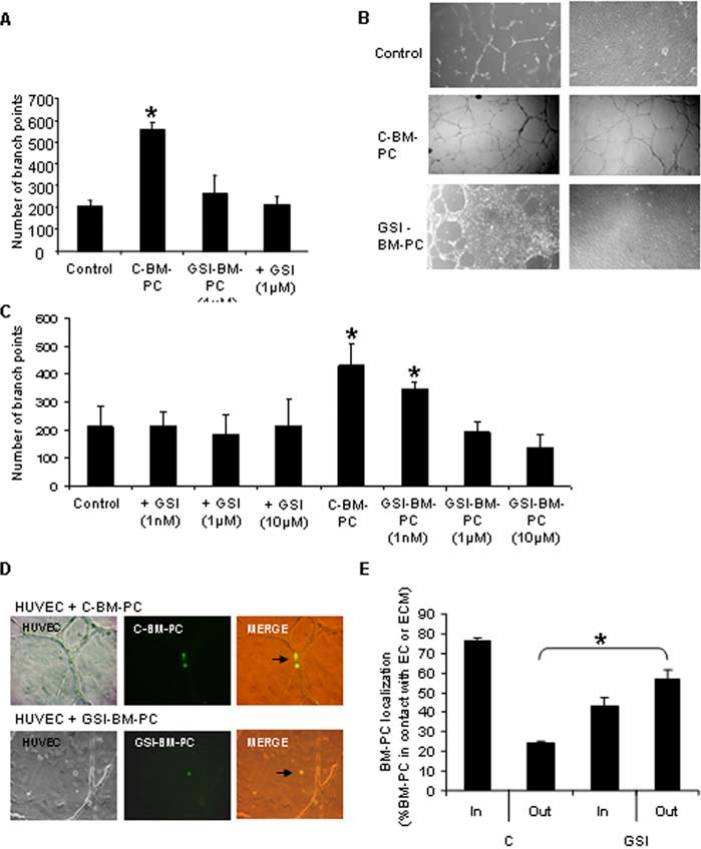
Notch pathway inhibition on BM-PC reduces their pro-angiogenic properties *in vitro*. A. Quantitative analysis of Matrigel-induced tube branching of HUVEC untreated or GSI-treated monoculture and co-cultured with control and GSI-treated BM-PC. Results show the average number of branch points in 5 high power fields. B. Representative images of HUVEC tube formation in monoculture and co-cultured with control and GSI treated BM-PC. Phase contrast microscopy (original magnification, 40×). C. Quantitative analysis of Matrigel-induced tube branching of HUVEC untreated or GSI-treated monoculture and co-cultured with control and GSI-treated BM-PC, using different doses of GSI treatment. D. Representative images of HUVEC tube structures in the presence of control or GSI BM-PC acetylated LDL-FITC labelled (original magnification, 40×). Arrows identify acetylated LDL-FITC labelled control or GSI BM-PC. E. Quantification of control or GSI BM-PC found in or out of endothelial tubular structures. Results expressed relatively to the total number of BM-PC counted. *P<0.05. Each experiment was performed in triplicate and the mean presented (n = 3).

After showing that Notch inhibition impairs BM-PC adhesion to ECM and their endothelial differentiation *in vitro*, we tested whether it also impaired their angiogenesis-stimulation capacity. As shown in Figure 4, endothelial cells co-cultured with control (untreated and DMSO treated) BM-PC for 18 hrs on Matrigel formed significantly more endothelial branches (quantified as branch points per high power field) than those resulting from endothelial cells co-cultured with GSI-treated BM-PC (the effect of GSI is dose-dependent, Fig 4 C). Since this pro-angiogenic effect of BM-PC could result from direct contact with endothelial cells or from paracrine (indirect) stimulation, we next quantified the number of BM-PC (labelled with ac-LDL) in contact with endothelial cells and those spread throughout the matrigel. As shown in Figure 4C and quantified in Figure 4D, the majority of untreated BM-PC are found in contact with the endothelial cells, in close proximity to branch points; in contrast, GSI-treated BM-PC are predominantly found throughout the matrigel (Fig 4D). Therefore, GSI treatment impairs the direct contact between BM-PC and endothelial cells. Importantly, the total number of BM-PC in contact with endothelial cells or adherent to the ECM is reduced by GSI treatment (not shown), highlighting the global role of Notch pathway in regulating BM-PC:endothelial cell adhesion and BM-PC:ECM adhesion.

### Notch pathway inhibition on BM-PC reduces their wound healing properties *in vitro*

**Figure 5:**
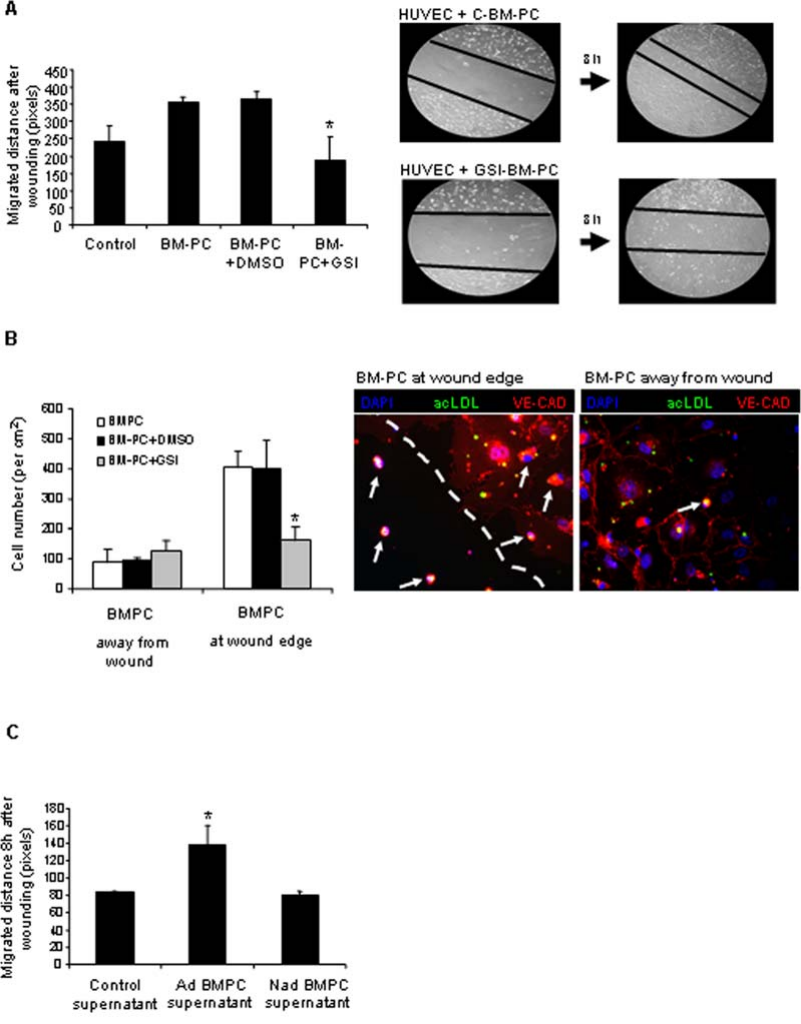
Notch pathway inhibition on BM-PC reduces their wound healing properties *in vitro*. Quantification of HUVEC migration during wound healing *in vitro*. HUVEC were cultured alone or in the presence of C/GSI treated BM-PC. Distance was measured in pixels using ImageJ software. Representative image of HUVEC at the beginning and at the end of the wound healing assay, in the presence of Control or GSI treated BM-PC. Phase contrast microscopy (original magnification, 200×). B. Quantitative analysis of Control and GSI treated BM-PC localization during HUVEC wound healing. BM-PC were classified has being on the wound site or over the HUVEC monolayer. Representative confocal image (×400) of BM-PC stained with acetylated LDL(FITC) and HUVEC (nuclear staining with DAPI). Dashed line represents the HUVEC wound edge. C. Quantitative analysis of HUVEC migration during wound healing assay. HUVEC were cultured with un-conditioned media or with conditioned media from adherent (Ad) /non-adherent (Nad) BM-PC. *P<0,05 Each experiment was performed in triplicate and the mean presented (n = 3).

Since treating BM-PC with GSI impaired their adhesion to ECM and their capacity to induce endothelial branching, next we asked whether it also inhibited their wound healing properties. As shown in Figure 5, the capacity to restore an endothelial monolayer (in a “wound healing” assay) is significantly improved upon the addition of control BM-PC to the wounded endothelial monolayer (Fig 5A). Control BM-PC predominantly adhered to the exposed ECM and to endothelial cells at the wound edge (Fig 5B). In contrast, GSI treatment reduced BM-PC adhesion to ECM and to the endothelial cells at the wound edge (Fig 5B). Importantly, supernatants obtained from adherent BM-PC also accelerated wound healing/endothelial monolayer recovery while supernatant obtained from non-adherent BM-PC failed to do so (Fig 5C). Taken together, these data suggest that BM-PC may promote wound healing by direct contact with endothelial cells at the wound edge and by adhering to the exposed ECM, but also that paracrine factor(s) released by the adherent BM-PC may stimulate endothelial cells during the wound healing process. These results also suggest that Notch pathway inhibition with GSI, by blocking BM-PC adhesion to ECM and activated endothelial cells, impairs BM-PC wound healing properties *in vitro*.

### Notch pathway modulation on BM-PC regulates their angiogenic and their wound healing properties *in vivo*

After showing that blocking the Notch pathway on BM-PC impairs their differentiation, adhesion to ECM, angiogenesis and wound healing promotion *in vitro*, next we tested the importance of these observations in a wound healing model *in vivo*. First, we verified that wounding induced mobilization of BM-PC to the peripheral blood of wounded mice (Figure S3).

**Figure 6:**
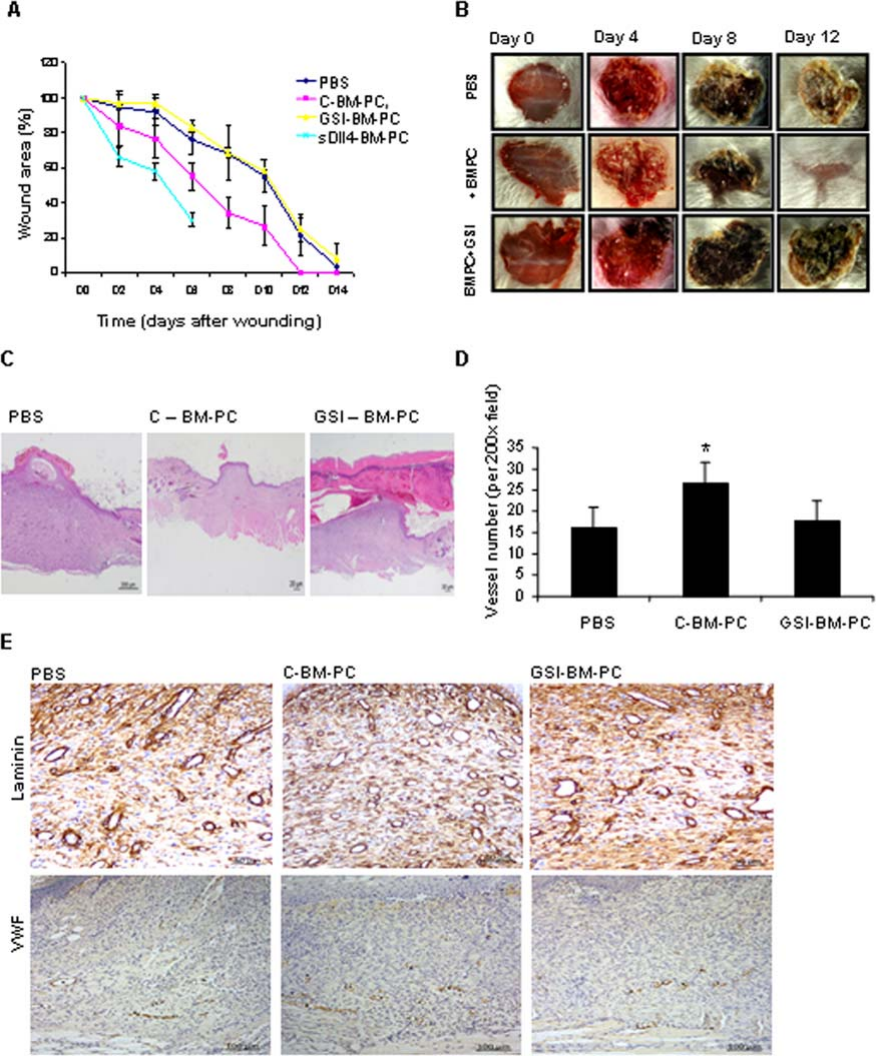
Notch pathway modulation on BM-PC regulates their angiogenic and their wound healing properties *in vivo*. A. Quantification of wound area in Balb-C mice injected with PBS, control BM-PC, GSI BM-PC or sDll4 BM-PC. Area at each time point is expressed relatively to the area measured immediately after wounding. B. Representative images of the wounds at days 0, 4, 8 and 12. C. Representative histological images of wounds collected at day 14 after wounding. Hematoxilin and Eosin staining. Scale bar represented. D. Quantification of vessel basement membrane immunostaining for laminin in the wound tissue of PBS, C-BM-PC or GSI BM-PC injected mice at day 14 post-wounding. E. Representative image of laminin and VWF immunostaining (* identifies VWF positive staining) in wounds of PBS, C-BM-PC or GSI BM-PC injected mice. Scale bar represented. *P<0.05 Each experiment was performed in triplicate and the mean presented (n = 3).

As quantified and shown in Figure 6A and B, injection of normal BM-PC to wounded mice improved wound healing significantly, while BM-PC pre-treated with GSI showed no effect (mice in this group showed similar rate of wound healing to PBS/non-injected mice). Importantly, the pro-wound healing property of BM-PC involved an angiogenesis response at the wound site. As shown and quantified in Figure 6D,E, mice injected with normal BM-PC showed a higher microvessel density at the wound site on days 7 and 14, while those that received BM-PC pre-treated with GSI showed a similar wound microvessel density to control (untreated) mice (Data shown for day 14, Fig 6D, E). The increase in angiogenesis at the wounds after BM-PC injection was detected using laminin as a microvessel basement membrane marker and von wilebrand factor as an endothelial marker (Fig 6 D,E) and also using desmin as a smooth muscle cell marker (data not shown). These results suggest that BM-PC stimulate endothelial sprouting *and* smooth muscle cell recruitment to the wound site. Taken together, these data suggest that Notch pathway inhibition with GSI impairs the capacity of BM-PC to promote wound healing and angiogenesis *in vivo*. In contrast, activation of the Notch pathway on BM-PC using soluble Delta-like 4 further improved their wound healing capacity (Fig 6A and Figure S4), strongly suggesting that Notch signalling pathway activation on BM-PC may be used to stimulate wound healing.

### GSI-treated BM-PC are found at lower frequencies in wound tissues

**Figure 7:**
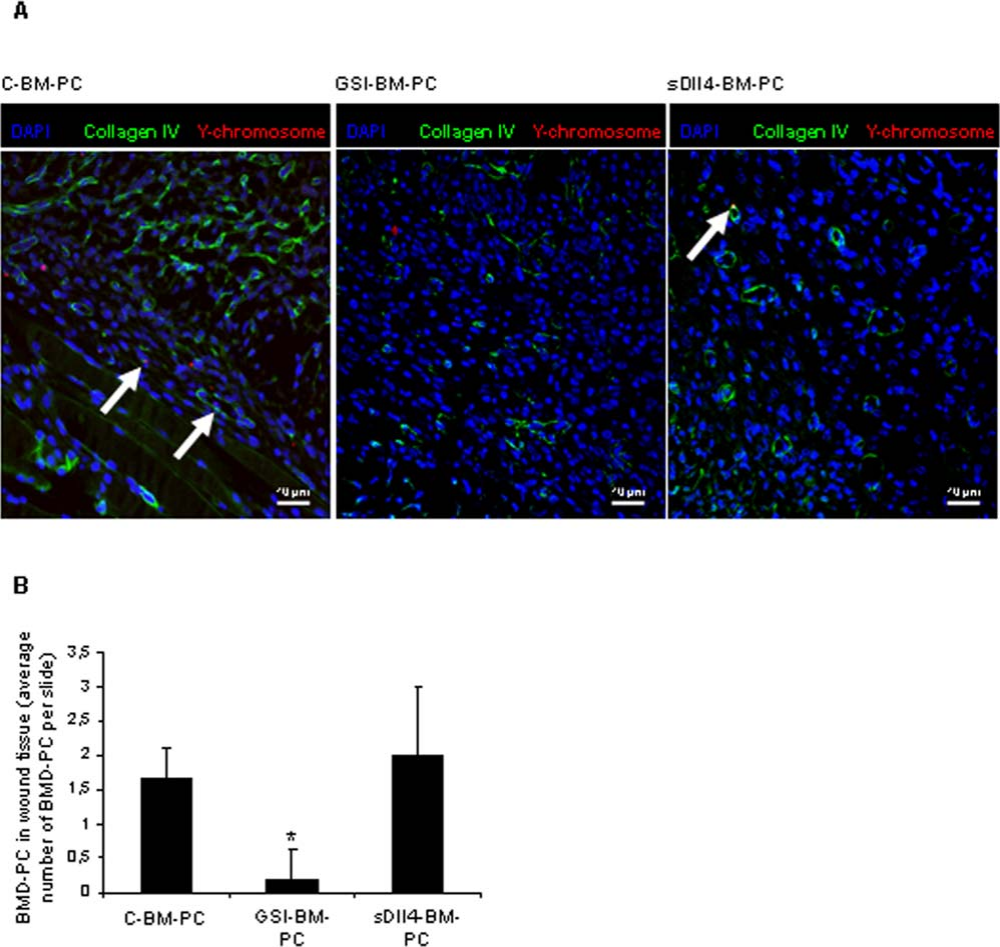
Control BM-PC are found at greater frequencies in wounds. A. Representative image of injected C, GSI – or sDll4 treated BM-PC at the wound site. BM-PC are identified as positive for Y-chromosome probe (white arrow). Immunostainning for collagen IV identifies vessel basement membrane. Scale bar represented. B. Quantification of BM-PC present at the wound. 10 slides per wound were used for BM-PC quantification. *P<0.05.

Having demonstrated that Notch pathway inhibition with GSI impaired the capacity of BM-PC to stimulate angiogenesis and to promote wound healing *in vivo*, we asked whether the frequency at which BM-PC are detected at the wound site might account for the differences observed. First, we verified that the number of BM-PC detected at the wound site on days 7 and 14 after wounding is very low (Fig 7 B). Nevertheless, as exemplified in Figure 7A, GSI treatment significantly reduced the frequency at which BM-PC are found in wound tissues. Moreover, as above, activation of the Notch pathway on BM-PC using soluble Delta-like 4 resulted also in higher numbers of BM-PC at the wound sites (Fig 7 B). These results suggest that Notch pathway inhibition by GSI, by impairing BM-PC adhesion to ECM and to endothelial cells in wounds, results in a lower frequency of BM-PC at the wound site and incorporated into neo-vessels.

## Discussion

A putative role for BM-derived progenitors in neo-vessel formation (angiogenesis) and vessel repair has been under intense scrutiny for the last decade. Numerous studies have argued that the contribution of this rare and heterogeneous cell population is essential for vessel activation and repair, although their precise function or the mechanisms involved remain elusive [22], [23]. Direct incorporation of BM-progenitor cells has been extensively shown in diverse models [24] but the low and variable frequency at which BM-progenitors are found incorporated into vessels is suggestive of an indirect (possibly paracrine or justacrine) role during the neo-angiogenesis processes. Therefore, it is of extreme importance to understand the mechanisms involved in the communication between BM-progenitors with angiogenic potential and endothelial cells at sites of neo-angiogenesis.

In the present work we used lin-sca1+ BM mononuclear cells (termed BM-PC throughout the manuscript), which under well defined pro-endothelial differentiation culture conditions [25] generate over 70–80% mature endothelial cells, to study their importance in angiogenesis and vessel repair during wound healing. Previous studies suggested that sca1+ cells are recruited into sites of vessel damage and ischemia and contribute to vessel healing and formation [26], [27].

We have previously characterized the gene expression profile of endothelial progenitors under pro-endothelial differentiation conditions [25], and observed the expression of members of the Notch pathway. In the present report, we exploited the hypothesis that this signalling pathway might be involved in the differentiation and, more importantly, in the function of BM-PC during neo-angiogenesis and vessel repair. The Notch pathway has been implicated in vasculogenesis in the embryo [14], [15], [16], [17], [18], as well as in adult tumor angiogenesis [28] and wound healing [19], although BM-derived progenitors were not studied in these settings. Considering that various components of the Notch pathway are expressed in BM-PC as well as in activated endothelial cells [29], we also explored the possibility that this pathway might promote the communication between the 2 cell types during physiological angiogenesis.

In the present work, we show expression of Notch1, Jagged 1 and Delta-like 4 on BM-PC and activation of Notch signalling during endothelial differentiation *in vitro*. We also demonstrate that inhibiting this pathway using the gamma secretase inhibitor (GSI) DAPT reduces the number of mature endothelial cells generated at the end of the differentiation assay. In addition, we reveal for the first time the importance of Notch pathway in BM-PC adhesion to different extracellular matrices. Treatment of BM-PC with GSI inhibited their adhesion to fibronectin, collagen, laminin and gelatin, suggesting this effect might be specific of BM-PC adhesion to the basement membrane. GSI treatment was shown to significantly reduce the expression of integrins alpha3 beta1 (which has affinity to the above mentioned ECM components), both at the transcriptional as well as protein level, and thus regulated BM-PC adhesion to the ECM. Moreover, siRNA against integrin alpha3 significantly reduced the adhesion of BM-PC during endothelial differentiation. There is some literature suggesting that the Notch pathway modulates integrin activity on endothelial cells [30] although only at the conformational level and not at the transcription and translational level. Interestingly, previous studies have shown that various components of the basement membrane are expressed in the vessel lumen during tumor [31] angiogenesis and also during wound healing [4]. These sites probably represent the preferential sites for BM-PC adhesion during vascular remodelling. Taken together, we suggest that BM-PC may interact with extracellular matrix exposed at sites of angiogenesis or vessel repair, and that the Notch pathway is involved in this interaction by modulating integrin expression. Although we cannot disregard other, off-Notch, effects in integrin modulation and BM-PC adhesion and differentiation, our data strongly suggests that this important signalling pathway is involved. In agreement, *in vitro* transfection of BM-PC with a constitutively active form of Notch 4 promoted their adhesion and augmented endothelial differentiation (Figure S2).

Next, we tested the role of the Notch pathway on the ability of BM-PC to induce endothelial cell activation, migration and tube formation. Notch inhibition with GSI reduced the capacity of BM-PC to stimulate endothelial tube formation *in vitro*, suggesting it affects their capacity to stimulate angiogenesis. In this tube formation assay, Notch pathway inhibition reduced the capacity of BM-PC to interact (incorporate?) with endothelial cells during angiogenesis *in vitro*. Since endothelial cells during angiogenesis and wound healing express members of the Notch pathway [29], we suggest this may be one mechanism by which BM-PC and activated endothelial cells interact.

Next, we tested the importance of the Notch pathway in the function of BM-PC in wound healing *in vitro* and *in vivo*. We demonstrate that normal BM-PC adhere to the ECM at the wound site and enhance endothelial migration and wound closure. On the other hand, GSI treatment reduced the adhesion of BM-PC to extracellular matrix, reduced their interaction with endothelial cells at the wound edge and failed to induce endothelial migration *in vitro*. Notably, supernatants collected from adherent BM-PC also improved wound healing, suggesting their adhesion may result in the production of pro-angiogenic growth factors such as VEGF, IL-8, among others that promote endothelial cell activation. Taken together these results show that Notch activity on BM-PC is necessary (via integrin modulation) for their ability to recognize and adhere to exposed ECM and activated endothelial migration. These results also suggest that the interaction between BM-PC and activated (wounded) endothelial cells is exerted in a direct/justacrine (Notch pathway) and indirect/paracrine fashion.

*In vivo*, intravenous injection of normal BM-PC in wounded mice increased angiogenesis at the wound site and improved wound healing, while pre-treatment with GSI reduced BM-PC homing, resulting in a decreased angiogenic response and delayed wound healing. These results imply that in the absence of Notch activation BM-PC lose their wound healing properties *in vivo*.

Inefficient cutaneous wound healing represents a serious medical challenge, namely in chronic wounds such as in diabetic [32], [33] and morbid obese patients [34]. Moreover, chronic or dysfunctional wound healing has been partially attributed to a lack of an appropriate vascular response [32], [33], and also to dysfunctional BM-derived endothelial progenitors [35], [36], [37]. Therefore, there has been considerable interest in modulating the vessels response during impaired wound healing for therapeutic purposes, namely via the use of BM-PC [38], [39]. In the present study, we reveal a crucial and previously undisclosed role of the Notch pathway in the function of BM-PC in angiogenesis responses during wound healing *in vitro* and *in vivo*.

**Figure 8:**
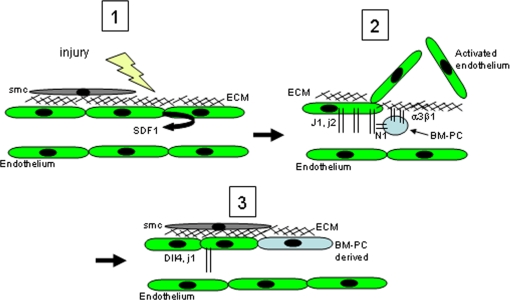
Proposed model of the mechanisms modulated by the Notch-Delta pathway on BM-PC during wound healing. 1. Wounded endothelial cells produce chemoattractant signals that recruit BM-PC into the wound site; 2. As a result of injury/wounding endothelial cells die, exposing extracellular matrix components in the vessel lumen; activated endothelial cells near the wound edge overexpress ligands of the Notch-Delta pathway, namely Jagged 1 and 2; BM-PC interact with endothelial cells at the wound site, and as a result of Notch-Delta activation these recruited cells overexpress integrin alpha3beta1, and bind the exposed extracellular matrix; as a result of BM-PC activation and adhesion, there is an angiogenesis induction (vessel sprouting) at the wound site. 3. Following angiogenesis activation and re-absortion of the wound tissue/scar, a small proportion of endothelial cells at the wound site derive from the recruited BM-PC, while the great majority derives from activated pre-existing endothelial cells. Following the pro-angiogenic response induced by BM-PC, vessel stabilization is promoted by recruitment of smooth muscle cells (desmin+).

Taken together, we propose a model which may explain the involvement of the Notch pathway in the function of BM-PC during wound healing (Figure 8): 1. Wounding promotes sca1+ BM-derived progenitor mobilization to the peripheral blood; 2. Activated endothelial cells at the wound site express Notch ligands, namely Jagged 1 and 2 [29] which may activate Notch signalling on circulating BM-PC; 3. Notch pathway activation on BM-PC up-regulates integrin alpha3beta1 and promotes BM-PC adhesion to extracellular matrix components at the wound site; 4. Adherent BM-PC stimulate endothelial activation (angiogenesis) in a justacrine and paracrine manner, resulting in improved wound healing.

We suggest that modulating the Notch pathway on BM-PC may be used to stimulate their wound healing potential, and further foster their use in chronic or delayed wound healing.

## Materials and Methods

The procedures involving mice were performed following Institutional (Instituto Gulbenkian de Ciencia) and National Guidelines. All experiments were approved by an Institutional Review Committee.

### BM-PC Isolation

To isolate BM-PC, four-to-eight-week-old male BALB/c mice were sacrificed and their bones collected in DMEM (Gibco) supplemented with 10%FBS (foetal bovine serum, Sigma-Aldrich, Germany). Bone-marrow was flushed-off using PBS with 2%FBS and then ficol (Histopaque-1077, Sigma Diagnostics, St. Louis, USA) was used to isolate total mononuclear cells (MNC). The lineage negative (lin-) fraction was isolated using mini-MACS immunomagnetic separation system (Mylteni Biotec, Bergish Gladbach, Germany), according to the manufacturer instructions, and was cultured overnight in RPMI 10%FBS with stem cell factor (Sigma-Aldrich, 1 ng/ml). Sca-1+ cell isolation was subsequently done using mini-MACS immunomagnetic separation system. Purity of the isolated cells was determined by FACS analysis using anti-Sca-1 antibodies (BD Pharmigen); isolated BM-PC were used in further experiments if their purity was above 95%.

### Cell Culture and Reagents

Isolated BM-PC were transferred onto 2% gelatine (Sigma-Aldrich), Fibronectin (10 µg/ml, Sigma-Aldrich), Colagen I (10 µg/ml, Cell Adhesion) or Laminin (10 µg/ml, Sigma-Aldrich) coated 24-well plates (1,5×10^5^ cells/well) and incubated in endothelial differentiation medium consisting of EBM-2 medium supplemented with 2% FBS, ECGS (20 µg/ml, Sigma-Aldrich), Heparin (5 U/ml, Sigma-Aldrich), VEGF (20 ng/ml, Sigma-Aldrich), HEPES buffer (ph = 7,5, 25 mM) and antibiotics.. Every 3 days the medium was supplemented with 1 µl VEGF (20 ng/ml) and 1 µl Heparin (5 U/ml). Around day 8 of differentiation non-adherent cells were washed off and new media was added. Cells were allowed to differentiate for 15–20 days under these conditions. RNA samples were collected at different time points and imunofluorescence staining was preformed at the end of the differentiation assay.

Human umbilical vein endothelial cells (HUVEC), passages 3 to 6, were cultured and maintained following standard procedures and culture conditions in complete EBM-2 medium (Clonetics). The γ-secretase inhibitor (DAPT, Sigma-Aldrich) was diluted in dimethyl sulfoxide (DMSO, Sigma-Aldrich) and used at a final concentration of 10 µM. In most experiments DMSO was used as control. Soluble Delta Ligand 4 (BD Pharmigen) was used at a concentration of 2 µg/ml.

### Plasmids, Antisense Oligonucleotides, and Cell Transfection

The plasmids bearing distinct forms of murine Notch4 were a gift from Tom Maciag Lab: constitutively active Notch 1 and Notch4 – CAN1 and CAN4 (C-terminal intracellular domain Int3 cloned into XhoI site of pcDNA3.1 hygro). Plasmid transfections were preformed using Lipofectamine (Invitrogen) accordingly with manufacturer's instructions. The antisense nucleotides (Applied Biosystems-Ambion) against integrin sub-units α3 (uuccgcugaaucauguacgtg) and β1 (ggauaaucayaguaauggctc) were used to transfect BM-PC using Oligofectamine (Invitrogen) accordingly with manufacturer instructions.

### Reverse-transcription Polymerase Chain Reaction (RT-PCR)

RNA extraction (Trizol, Invitrogen), cDNA synthesis (Reverse-transcription with Superscript II reverse transcriptase (Invitrogen)) and oligo (dT) primer (Roche) and RT-PCR were performed following standard protocols. Primers used in the RT-PCR reactions were mHes1 (tctacaccagcaacagtgg; tcaaacatctttggcatcac), mHey1 (tgagctgagaaggctggtac; accccaaactccgatagtcc), mHey2 (tgagaagactagtgcaacag; tgggcatcaaagtagccttta), mNotch1 (cggtgaacaatgtggatgct; actttggcagtctcatagct), mNotch4 (attgaattcggataaagatgcc; agcgttagcaggtcccagtgac), mDll4 (ctgtccttatggctttgtgg; gctccttcttctggtttgtg), mDll1 (acagaaacaccagcctccac; gccccaatgatgctaacaga), mJagged1 (ccagccagtgaagaccaagt; tcagcagaggaaccaggaaa), mJagged2 (gaggtcaaggtggaaacagt; tgtccaccatcagcagataa), mITGA3 (tgtgtacctgtgtcccctca; atgccggtctgcaagtagtc), mITGB1 (ccaaatcttgcggagaatgt; cattcatcaaatccgttcca). The housekeeping gene used to normalize the samples was mß-actin (agccatgtacgtagccatcc; ctctcagctgtggtggtgaa). Each sample was analyzed in duplicate and each PCR experiment included at least one non-template control well. PCR products were electrophoresed through 2% agarose gel and analyzed by staining with ethidium bromide.

### Immunofluorescence

Cells were fixed in 2% paraformaldehyde for 15 minutes at 4°C, blocked with PBS+0,1% BSA for 45 minutes at room temperature and incubated with primary antibody overnight (diluted in PBS+0,1% BSA+0,1% Triton X-100). Antibodies used were von Willebrand Factor (vWF, 1∶200, A0082, Dako, Germany), Flk-1 (5 µg/ml, AF644, R&D Biosystems), CD31 (1∶100, 553370, BD Pharmigen), P-H3 (1∶100, 06-570, Upstate – Cell Signaling Solutions), VE-cadherin (1∶100, sc-6458, Santa Cruz Biotechnology), integrin α3 (1∶100, sc-7019, Santa Cruz Biotechnology) and integrin β1 (1∶100, AF2405, R&D systems, Inc.). For LDL incorporation cells were cultured in FITC-conjugated acetylated LDL (ac-LDL, 1∶1000, L23380, Invitrogen – Molecular Probes) during 4 h before fixation. Secondary antibodies used: anti-rat/goat/rabbit FITC/PE-coupled IgG (Alexa fluor 488/594, Molecular Probes, US). Cells were examined by standard fluorescence microscopy using a fluorescence microscope (Axioplan Microscope, Zeiss, Germany).

### Adhesion Assays

Isolated BM-PC were transferred onto 2% gelatine, Fibronectin (10 µg/ml), Colagen I (10 µg/ml) or Laminin (10 µg/ml) coated 24-well plates (1,5×10^5^ cells/well) and incubated in complete EBM-2 medium. 48 h after seeding, non-adherent cells were removed (using sterile PBS) and adherent cells were counted in 6 random high power fields (×200). The number of adherent cells was normalized relatively to the control (gelatine) condition.

### *In Vitro* Wound Healing Assays

HUVEC were harvested by brief trypsin digestion and seeded at a density of 5×10^4^ cells per cm^2^ on a 24-well plate, allowed to grow to a confluent monolayer, and then a scratch wound with a yellow tip (0,1 mm in diameter) was made at the length of the plate. After the scratch, the wells were rinsed with PBS to remove detached cells and EBM-2 medium (2%FBS) was replaced. To determine the effect of BM-PC on HUVEC monolayer recovery/wound healing, 1,5×10^5^ BM-PC untreated or treated with DAPT were added. To determine the effect of secreted factors by adherent or non-adherent BM-PC in wound healing we added their conditioned medium (collected after 24 h) to wounded HUVEC, using EBM-2 alone as a control. The total distance migrated by wounded HUVEC was evaluated using computer image analysis (NIH Image J analyzer) and expressed as percentage of control (without BM-PC). The distance between the wound edges was measured immediately after wounding and 8 h later. The difference between the 2 measurements was considered as the total distance migrated by the wounded HUVEC. Adherent BM-PC quantification was obtained by counting the number of BM-PC at the wound site (adherent to exposed extracellular matrix or to HUVEC at the wound edge) versus the number of BM-PC adherent to HUVEC away from the wound. Data is represented relative to the wound or monolayer area.

### *In Vitro* Tube Formation Assay

HUVEC were seeded on Matrigel (BD Bioscience) – coated wells (24 well plate) at a density of 1×10^5^ cells per well in EBM-2 medium (2%FBS). Untreated, DMSO and DAPT treated BM-PC were added at a density of 1,5×10^5^ cells and then incubated for 16–18 h at 37°C. To determine the localization of BM-PC during the assay, we first incubated these with FITC-conjugated ac-LDL for 4 h to allow further visualization. After endothelial cell tube formation was observed the cells were fixed in paraformaldehyde (2%). Photographs were taken at 10× and 20× magnification using an Olympus Microscope. Branch quantification was done using the NIH Image J analyzer and expressed as a percentage of the control condition (without BM-PC).

### *In Vivo* Wound Healing Model and Wound Closure Analysis

*In vivo* wound healing model was established using Balb-SCID mice. Briefly, female mice, 8 weeks old, body weight 20–33 grams, were anesthetized with intraperitoneal injection of a combination of xylazine (10 mg/kg) and ketamine (100 mg/kg). After shaving the hair, 2 single full thickness, 6-mm diameter excisional wounds were performed in the dorsolumbar skin with a sterile biopsy punch. Mice were individually caged. BM-PC were injected in the tail vein on the day of wound infliction (day 0) and at day 4 post-wounding. For each injection 2,5×10^5^ BM-PC untreated or treated with DAPT or sDll4 were used. Photos were taken every 2 days starting on day 0 and wound area was calculated (Π.r1.r2). Wound area at each time point was represented relatively to the area obtained at day 0.

### Wound histology and Immunohistochemistry

Animals were sacrificed at days 7 and 14 post-wounding. 8 mm diameter skin biopsy samples centred on the wound bed were collected, fixed in 10% formalin for a maximum of 48 hours and embedded in paraffin. Wounds were serially sectioned (3 µm) perpendicular to the wound surface, rostral to caudally, with a 500 µm intermission, and stained with haematoxylin and eosin (H&E). The number of levels analysed ranged from 8–10 per wound.

To visualize blood vessels, sections adjacent to those stained for H&E were labelled for vWF(1∶300, A0082, Dako) and laminin (1∶200; L9393 Sigma-Aldrich, Germany). Briefly, sections were deparaffinised and immersed in methanol with 0.3% hydrogen peroxide for 30 minutes. Antigen retrieval was achieved in protease K for 30 minutes, followed by blocking with 0.1% BSA in PBS and overnight incubation with the primary antibodies. Immunolocalization was achieved using biotinylated swine anti-rabbit IgG antibody (Dako) and peroxidase-conjugated streptavidin, 30 min each, and visualized with DAB (Dako) counterstained with Mayer's hemalumen (Merck, Germany).

Microvessel density (MVD) was evaluated through laminin and vWF immunoreactivity. At low power field (×40), tissue sections were screened and 5 areas with the most intense neovascularization (hot spots) were selected. Microvessel counts of these areas were performed at high power field (×200). The mean microvessel count of the five most vascular areas was taken as the MVD, which was expressed as the absolute number of microvessels per 0.74 mm2 (×200 field).

### ImunoFISH detection of transplanted BMD-VPCs within wound sections

Wound sections were deparaffinised and antigen retrieval was achieved in 0,01 M sodium citrate buffer followed by 15 minutes Pepsin 0,4% digestion. Sections were immunostained for Colagen IV (1∶100, AB769, Chemicon International), and secondary antibody anti-goat-Alexa 488. Following immunoflorescence, the sections were hybridized with a probe against the Y chromosome (Cambio, UK) using a denaturation temperature of 75–80°C for 5 minutes and hybridization temperature of 37°C overnight.

### Statistical Analysis

Differences between the experimental groups (cell numbers, migrated distances, wound size among other parameters) were calculated using ANOVA or T student test.

## Supporting Information

Figure S1 Differentiated BM-PC form tubes on Matrigel. A. Quantification of tube formation on day 0 or day 20 BM-PC plated or matrigel for 16 h; *P<0.05 Each experiment was performed in triplicate and the mean presented (n = 3).
(0.16 MB TIF)

Figure S2 Constitutively active Notch 4 activates the Notch pathway on transfected BM-PC, promotes their adhesion. A. Activation of the Notch pathway, as shown by expression of downstream targets, on BM-PC transfected with constitutively active Notch 4. B. Constitutively active Notch 4 increases BM-PC adhesion during in vitro endothelial differentiation.
(0.06 MB TIF)

Figure S3 Wounds induce mobilization of sca1+ cells in vivo. A, Quantification of Sca-1+ cells in the peripheral blood of wounded Balb-SCID mice. Results represented relatively to control/not wounded at given time points.
(0.05 MB TIF)

Figure S4 Pre-treatment of BM-PC with soluble Dll4 induces expression of Notch target genes. A. BM-PC pre-treated with soluble Dll4 show evidence for transcription of Notch pathway downstream targets.
(0.05 MB TIF)
