# Supplementary figures and images for: Notch Pathway Modulation on Bone Marrow-Derived Vascular Precursor Cells Regulates Their Angiogenic and Wound Healing Potential
Source: PLoS One. 2008 Nov 18;3(11):e3752. doi: 10.1371/journal.pone.0003752 (PMC2582964; doi:10.1371/journal.pone.0003752)

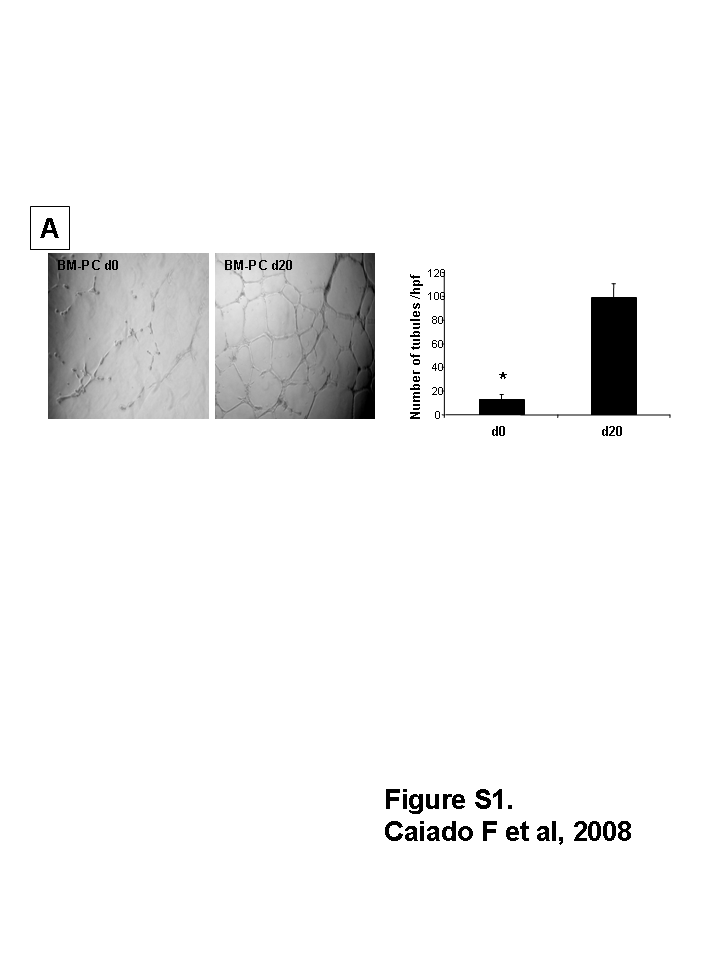

Supplement: Figure S1 — Differentiated BM-PC form tubes on Matrigel. A. Quantification of tube formation on day 0 or day 20 BM-PC plated or matrigel for 16 h; *P<0.05 Each experiment was performed in triplicate and the mean presented (n = 3). (0.16 MB TIF) [file pone.0003752.s001.tif]

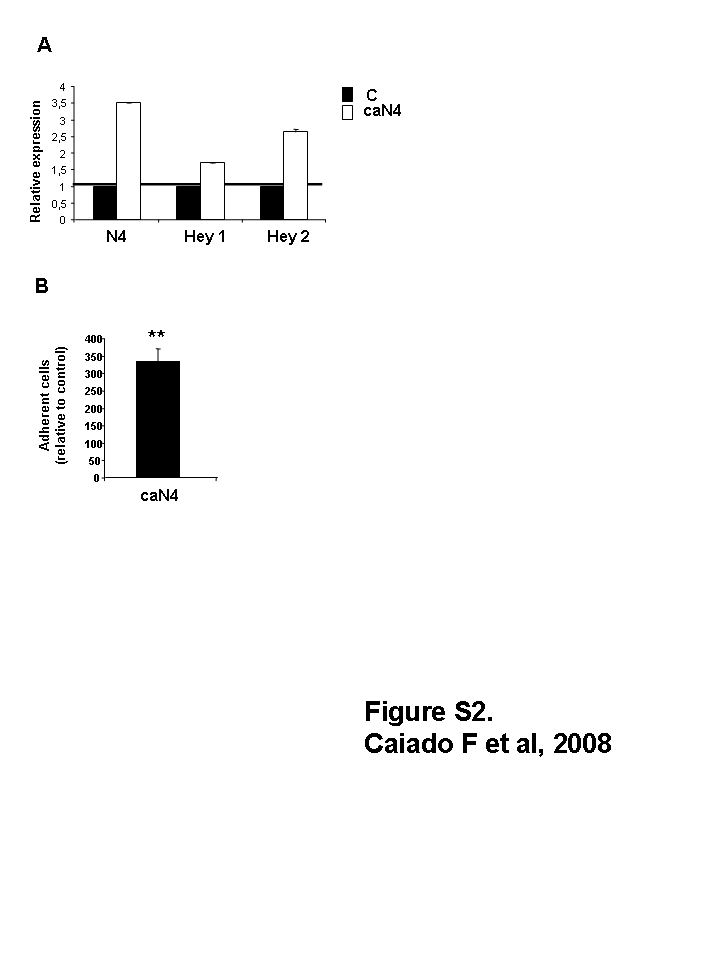

Supplement: Figure S2 — Constitutively active Notch 4 activates the Notch pathway on transfected BM-PC, promotes their adhesion. A. Activation of the Notch pathway, as shown by expression of downstream targets, on BM-PC transfected with constitutively active Notch 4. B. Constitutively active Notch 4 increases BM-PC adhesion during in vitro endothelial differentiation. (0.06 MB TIF) [file pone.0003752.s002.tif]

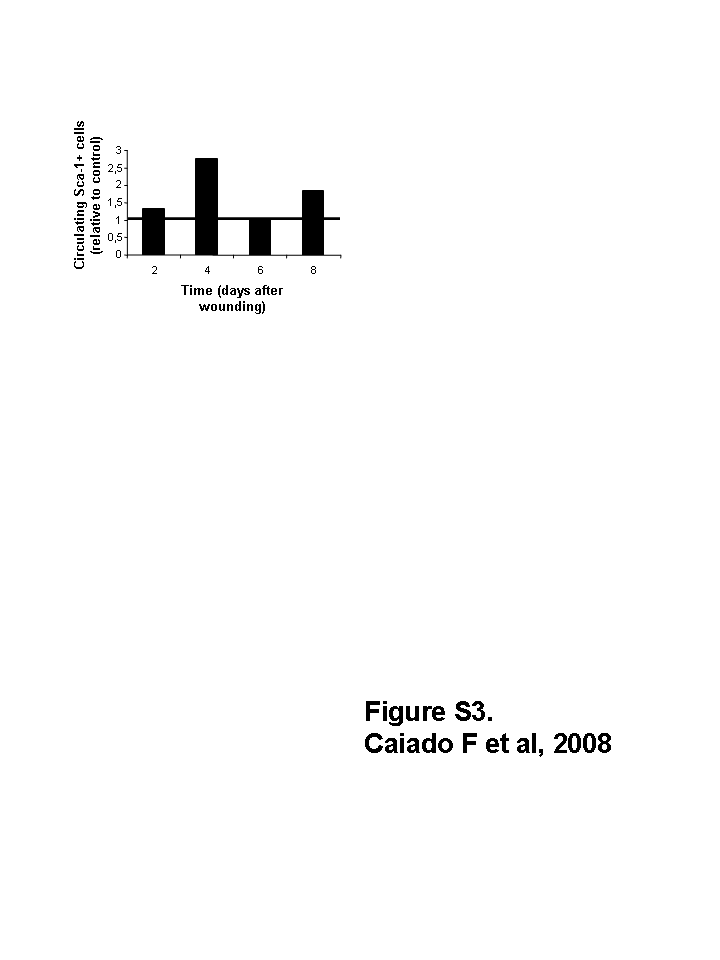

Supplement: Figure S3 — Wounds induce mobilization of sca1+ cells in vivo. A, Quantification of Sca-1+ cells in the peripheral blood of wounded Balb-SCID mice. Results represented relatively to control/not wounded at given time points. (0.05 MB TIF) [file pone.0003752.s003.tif]

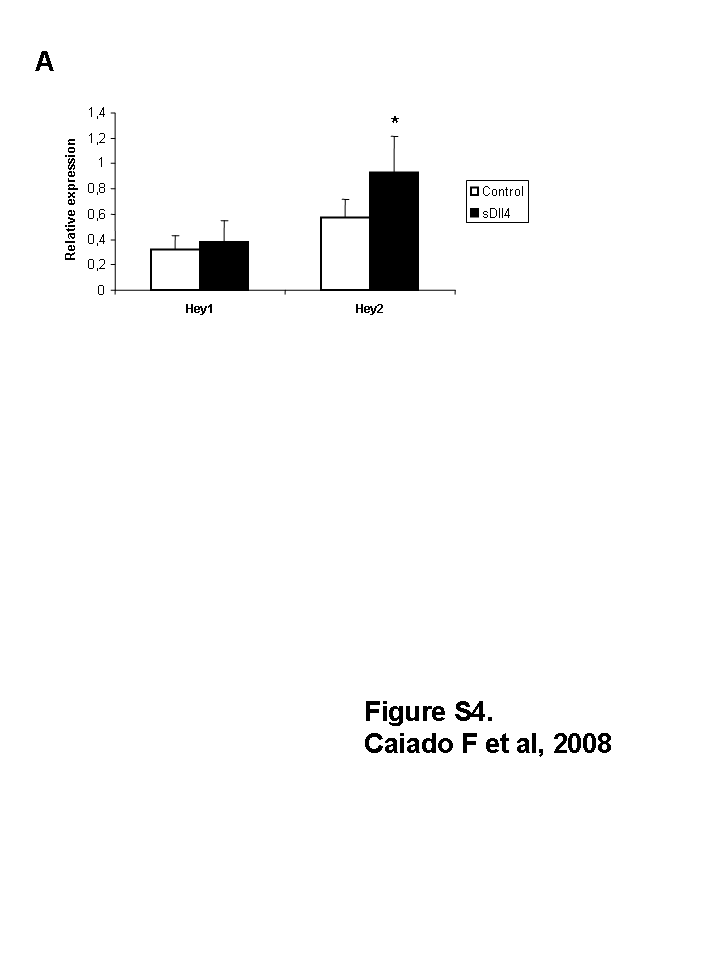

Supplement: Figure S4 — Pre-treatment of BM-PC with soluble Dll4 induces expression of Notch target genes. A. BM-PC pre-treated with soluble Dll4 show evidence for transcription of Notch pathway downstream targets. (0.05 MB TIF) [file pone.0003752.s004.tif]
